# Selective Blood Cell Hitchhiking in Whole Blood with Ionic Liquid-Coated PLGA Nanoparticles to Redirect Biodistribution After Intravenous Injection

**DOI:** 10.21203/rs.3.rs-3146716/v1

**Published:** 2023-07-11

**Authors:** Christine M. Hamadani, Gaya S. Dasanayake, Claylee M. Chism, Meghan E. Gorniak, Wake G. Monroe, Anya Merrell, Mercedes C. Pride, Rebekah Heintz, Karen Wong, Mehjabeen Hossain, George Taylor, Sara X. Edgecomb, Deauntaye Jones, Joy Dhar, Alison Banka, Gagandeep Singh, Priyavrat Vashisth, Joh'nis Randall, Donovan S. Darlington, Jaylon Everett, Ethan Jarrett, Thomas A. Werfel, Omolola Eniola-Adefeso, Eden E. L. Tanner

**Affiliations:** University of Mississippi; University of Mississippi; University of Mississippi; University of Mississippi; University of Mississippi; University of Mississippi; University of Mississippi; University of Mississippi; University of Mississippi; University of Mississippi; University of Mississippi; University of Mississippi; University of Mississippi; University of Mississippi; University of Michigan; University of Mississippi; University of Mississippi; University of Mississippi; University of Mississippi; University of Mississippi; University of Mississippi; University of Mississippi; University of Michigan; University of Mississippi

## Abstract

Less than 5% of intravenously-injected nanoparticles (NPs) reach destined sites in the body due to opsonization and immune-based clearance in vascular circulation. By hitchhiking *in situ* onto specific blood components post-injection, NPs can selectively target tissue sites for unprecedentedly high drug delivery rates. Choline carboxylate ionic liquids (ILs) are biocompatible liquid salts <100X composed of bulky asymmetric cations and anions. This class of ILs has been previously shown to significantly extend circulation time and redirect biodistribution in BALB/c mice post-IV injection via hitchhiking on red blood cell (RBC) membranes. Herein, we synthesized & screened 60 choline carboxylic acid-based ILs to coat PLGA NPs and present the impact of structurally engineering the coordinated anion identity to selectively interface and hitchhike lymphocytes, monocytes, granulocytes, platelets, and RBCs in whole mouse blood for *in situ* targeted drug delivery. Furthermore, we find this nanoparticle platform to be biocompatible (non-cytotoxic), translate to human whole blood by resisting serum uptake and maintaining modest hitchhiking, and also significantly extend circulation retention over 24 hours in BALB/c healthy adult mice after IV injection. Because of their altered circulation profiles, we additionally observe dramatically different organ accumulation profiles compared to bare PLGA NPs. This study establishes an initial breakthrough platform for a modular and transformative targeting technology to hitchhike onto blood components with high efficacy and safety in the bloodstream post-IV administration.

## INTRODUCTION

### Cell-selective ionic liquid-coated polymeric nanoparticles may overcome intravenous drug delivery barriers in systemic circulation for more efficient organ targeting.

Despite their potential for targeted and non-toxic delivery of systemically-administered drugs, particularly chemotherapeutics^1-5^, very few intravenous (IV) nanoparticle (NP) delivery systems, and no polymer-based NPs, have successfully made it into the clinic^6^. A primary challenge remains the premature clearance of polymeric nanoparticles by the immune system soon after intravenous (IV) injection, initiated by mononuclear phagocytic system (MPS) components within the blood^7,8^. The mechanism of this clearance is driven by opsonization–the process of serum proteins rapidly adsorbing to NP surfaces and triggering the immune system via a series of monocyte and protein-signaling cascades^9-12^. In addition to phagocytosis, this system shuttles NPs to off-target accumulation sites, such as the kidneys, spleen, and liver, acting as filtration systems for rapid clearance^13,14^. This creates a significant challenge in designing bioresponsive and targeted IV NP systems, as, despite the surface functionalization strategy, fewer than 5% currently reach their destination after IV injection^15-18^. Hydrophilic PEGylation and lipid nanoparticles (LNPs) are two current strategies to extend circulation half-life time. However, most (~60%) patients are prone to develop anti-PEG antibodies in their bloodstream^19-22,^ and PEGylation was previously shown to drive increased particle phagocytosis by neutrophils in human blood^123^. Additionally, depending on their formulation, LNPs are limited by range of targeting selectivity, sensitive storage conditions, instability in-vivo, and acute immune responses associated with toxicity caused by recent ionizable lipid technology^23-25^. Re-engineering of NP bio-interactions in the bloodstream via cellular hitchhiking is a new and recent approach that allows for biocompatible and facile re-directing of systemic NPs to desired tissues without significant interaction with the MPS ^26-31^. However, these bioinspired systems are also limited as they involve extraction of blood cells, combination, and re-injection back into the bloodstream, creating a challenge for streamlining accessible clinical translation ^32^.

Ionic liquids are liquid salts under 100 °C, composed of bulky and asymmetric cations and anions, which have novel potential to act as drug delivery biomaterials, seen in transdermal, antimicrobial, transbuccal, oral, and intravenous systems ^33-43^. With high structural tunability of either cationic or anionic partner, the resulting physicochemical and biophysical properties can be controlled both within the IL and with its interfacing environment ^44-46^. Furthermore, changing the length in alkyl chain structure of the cation of the IL has been shown to modulate interactions with albumin^47^, a primary serum protein in the bloodstream that participates in the protein adsorption and opsonization process^48^. A choline and trans-2-hexenoate IL has been used to electrostatically coat PLGA surfaces in order to repel serum proteins as well as direct affinity to RBC membranes in situ, thereby significantly extending circulation half-life and redirecting biodistribution to the first encountered capillary bed post-IV injection in both BALB/c mouse and Sprague-Dawley rat in vivo models^49-50^. We rationalize that by engineering the anion identity around that of trans-2-hexenoic acid in choline carboxylate ILs, we can identify new analog structures [ seen in Scheme 1] that drive selective affinity to white blood cell subpopulations, platelets, as well as RBCs, in whole blood. By engineering cellular hitchhiking affinity in whole blood, the targeting for diseased tissue sites can be significantly enhanced in an unprecedented way for virtually any disease manifesting at those locations, and in systemic circulation.

### Ionic liquid engineering and nanoformulation of IL-PLGA NPs for redirected biodistribution via cellular hitchhiking *in situ* post-intravenous administration

Herein, we have synthesized and screened a library of biocompatible choline carboxylic acid-based ILs to coat PLGA nanoparticles, whereby we have structurally engineered the anion’s alkyl chain length, saturation degree, functionalization moieties (methyl and oxygen groups), and the cation: anion ionic ratio. We have physicochemically characterized the IL-coated particles (IL-NPs) by Dynamic Light Scattering (DLS), quantitative ^1^H NMR spectroscopy, and fluorescent encapsulation efficiency (%EE), and studied their biophysical interactions in whole mouse and human blood ex vivo, and their pharmacokinetic and biodistribution profile in vivo. We first synthesized carboxylic acid-terminated poly(lactic-co-glycolic) acid (PLGA [50:50])-based nanoparticles (NPs) via nanoprecipitation and solvent evaporation using an acetonitrile (ACN)-based organic phase with the far-red fluorescent dye 1,1'-dioctadecyl-3,3,3',3'- tetramethylindodicarbocyanine, 4-chlorobenzenesulfonate (DiD, 2% wt/wt polymer) ^51^. Via DLS, bare DiD-loaded particles yielded a diameter of 57.83 5.61 nm, zeta potential of −22.94 3.85 mV, and PDI of 0.19 0.07 (n=4). We then synthesized ~60 different choline carboxylate ILs, which were coated on PLGA NPs and yielded a range of sizes below and above 200 nm, albeit with consistent anionic surface charge shifts and overall low PDI (< 0.3) (Table S1).

Bare & IL-coated NPs (with saline control) at 1 mg/mL were screened for hitchhiking potential by first being mixed with whole commercial BALB/c mouse blood (Bio-VT, NY, USA) at a 1:10 (v/v) ratio, approximately 100 NPs: RBC. After a brief mechanical inversion and 20 minutes of shaking incubation at 37 °C, the blood was centrifuged at 1000xg for 10 minutes, and each layer -- serum, white blood cells (WBCs), platelets, & red blood cells (RBCs) – was isolated carefully by micropipette using its cellular density gradient and placed into 1.5 mL microcentrifuge Eppendorf tubes, which were washed 3x with 1x PBS (pH 7.4) at 200xg for 5 minutes to remove unbound NPs. WBCs, platelets, and RBCs were then analyzed by Fluorescent Activated Cell Sorting (FACS) to identify the fraction of gated singlet cells colocalized with the NPs’ far-red dye.

**Figure 1:**
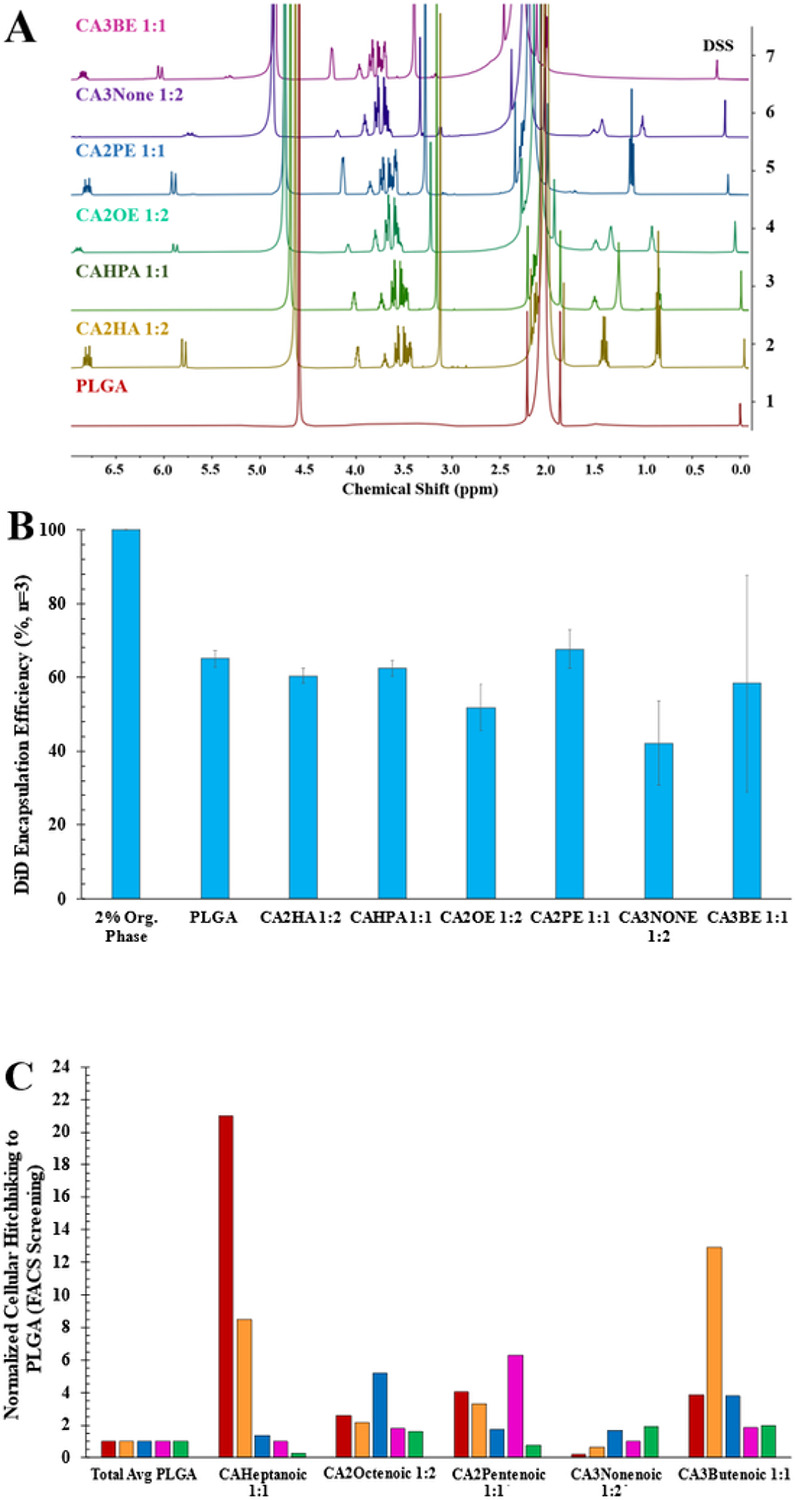
IL-PLGA DiD NP top candidates demonstrate unique chemical compositions when coating PLGA NPs, varying but functional encapsulation efficiencies (EE, %), and magnitudes-greater hitchhiking affinities towards specific cell-types vs. PLGA NPs in whole blood. **A)** Quantitative ^1^H NMR spectroscopy (DSS, 0.00 ppm: 0.2 mg DSS/1 mg NPs in D_2_O) illustrates the impact of varying carboxylate anion structure (violet-gold) on the assembly of PLGA NP surfaces (red), quantified in Table S3. **B)** DiD Encapsulation Efficiency (EE%) by fluorescent plate reader shows the impact of each top candidate IL coating on the fluorescence of PLGA DiD NPs, ranging from 40-70%, when directly compared to the organic phase used for synthesis (Table S4). **C)** From a FACS screen of 60+ screened IL-PLGA DiD NPs in whole blood (normalized to bare PLGA DiD NPs), top IL candidates show unprecedented and selective cellular hitchhiking to RBCs (red), platelets (orange), lymphocytes (blue), monocytes (pink), and granulocytes (green).

After screening ~60 candidates by FACS, a structure-function relationship was found to drive affinity to certain cellular subpopulations within whole blood compared to others (Figure 1 and Fig.S1A&B). Particularly, anion chain length, saturation, and molar ionic ratio between cation: anion all play a role in redirecting cellular affinity in whole blood (full ranked heatmaps available in Fig.S1). For instance, with regard to RBCs (Fig.1C), saturated anions at a 1:1 ratio demonstrated a clear increasing approach towards a 7-carbon hotspot (~21.0); however, they showed no affinity from 8-10 carbon chain lengths. This may be due to stronger hydrophobic interactions possible with the longer saturated chains reducing the IL-NPs’ protein avoidance. Consistent with this theory, saturated anions at 1:2 maintained singular affinity at 5 carbons (15.6) with worse performance across the 6-10 carbon chains. Methylating the second carbon for saturated chains at 1:1 decreased their respective overall affinity but shifted the hotspot from 7 carbons to 6 carbons (7.8), favoring shorter chains. At a 1:2 cation: anion ratio, 2-methyl hexanoic acid maintained the best RBC performance (7.1).

While we previously demonstrated the RBC-hitchhiking capabilities of CA2HA 1:2, structural screening analysis conducted in this report concurred that adding a trans double bond to the anion generally increased its hitchhiking capability, but that placement drastically affected performance (notably, 8-carbon chains consistently performed the worst for RBCs in every structural category but directed the highest lymphocyte affinity when in the 2-unsaturated chains in Fig.S1E). For instance, at a 1:1 ratio, C2-unsaturation maximized performance at the shortest chain length (CA2BE 1:1, 17.6), drastically decreased at 5 carbons, and again increased from 6 carbons to 9 carbons (11.0). However, at a 1:2 ratio, 2-unsaturated anions all performed roughly the same (4-7 carbons, 9 carbons: ~6-11). In comparison, at a 1:1 ratio, 3-unsaturation showed a steep increase approaching the 6-carbon hot spot (15.1), with loss of affinity as the chain is increased to 9 carbons. However, at a 1:2 ratio, 3-unsaturation shifted the hotspot to 5 carbons (6.7), albeit reduced in comparative RBC affinity compared with the 1:1 ratio. When methylating the 2-unsaturated anions at the C2 position, RBC affinity was comparatively quenched relative to the unmethylated anion analogue, with redirection of CA2Me2PE affinity to lymphocytes. Interestingly, at a 1:2 ratio, methylating trans-2-pentenoic acid uniquely enhanced its RBC affinity (19.0). Hence, at a 1:1 ratio, the methylation position requires being staggered with the double bond with one degree of separation to enhance RBC affinity, while at 1:2, methylation enhances RBC affinity when on the same carbon position as the double bond.

In contrast, platelet hitchhiking affinity (Fig.S1D) followed almost the opposite relationship to RBCs. For instance, for 2-unsaturated carbon chains, a gradient relationship was observed between 4-8 carbons, but the hot spot was at 9 carbons for 1:1 (5.1) and at 7 (13.2) and 9 carbons (13.3) for 1:2, illustrating the longer carbon chains required for higher platelet affinity when the double bond is present at C2 (as opposed to the short-medium length end of the spectrum for RBCs). However, more rigid structures may perform best for platelet hitchhiking at shorter alkyl lengths: when the 2^nd^ carbon is methylated on saturated anions, the 1:1 ratio shows a shift in hotspot to 6 carbons (CA2MeHexa 1:1), and to 5 carbons (CA2MePenta 1:2) at a 1:2 ratio, shorter than the 7 carbon (CAHPA 1:1) hotspot observed for RBCs. Additionally, while RBC affinity at 3-unsaturation is concentrated around medium-chain length anions, platelet hitchhiking shows the highest and most selective affinity at the most rigid and shortest length anion (CA3BE 1:1), drastically contrasted at its opposite relationship at 1:2 (highest affinity from 8-10 carbons).

Interestingly, screened candidates for granulocyte hitchhiking demonstrated either high but non-specific affinity or lower affinity and higher specificity. However, granulocytes showed the highest structural location-based contrast compared to monocyte hitchhiking (Fig.S1F and Fig.S1G). For example, shorter unsaturated structures CA3Me2HA 1:2 and CA2PE 1:1 (CA2PE 1:1 selected as the top candidate due to higher biocompatibility) performed the most selectively for monocytes, while longer unsaturated structure CA3None 1:2 comparatively had slightly less affinity than CA2None 1:2 or CA3DE 1:2 but was the most selective to granulocytes amongst the screened candidates when normalized to PLGA. Because of its selectivity, CA3None 1:2 was thus chosen as a top candidate to show proof-of-principle. However, if multiple targets were desired, other highly performing screened candidates could be chosen for study.

The DLS profile of the selected top in-situ hitchhiking candidates (Fig.1C) successfully remained under 200 nm within a size range of 130-170 nm, with surface charge ranges between −49 & −65 mV, and 0.06-0.20 PDI range (Table S2). We then studied the IL coating assembly using quantitative ^1^H NMR spectroscopy (Fig.1A). We were able to resolve anionic protons (5.5-7.0 ppm) reflecting the degree of alkyl chain unsaturation (violet, indigo, blue, green, and fully saturated gold), and we noted the formation of new multiplet patterns between choline peaks (3.5-4.0 ppm). This evidences a unique choline-PLGA surface “footprint” for each IL-PLGA coating assembly, while the negative surface charge indicates that the anion interfaces with the environment on the outermost layer of the coating (Table S1, Surface Charge). Additionally, we used a deuterated sodium trimethylsilylpropanesulfonate (DSS) standard to quantify the precise amount of total IL/mg PLGA and the molecular weights of the synthesized ILs, which allowed us to calculate a 24-hour IC50-biocompatible range ^52-56^ of approximate IL dosages (4.90 x 10^−7^ to 3.50 x 10^−6^ mol IL/100 μL dose for a ~25 mg mouse) for in-vivo administration (Table S3). Interestingly, anion hydrocarbon length played both a factor in the size of the coated nanoparticles (Table S2), as well as the amount of IL on the NP (Table S3), indicating that the bulkiness of the structure and likely sterics (from the location placement of double bond) played a role in the preferential assembly dynamics during nanoparticle coating. For example, the least number of molecules of the 9-carbon chain CA3None 1:2 (granulocyte candidate) was required (4.90 x 10^−7^ mol/mg PLGA) to created one of the largest coated NPs (164.1 37.2 nm), while the 5-carbon chain CA2PE 1:1 (monocyte candidate) required the highest number of molecules (3.50 x 10^−6^ mol/mg PLGA) at the lower range of IL-NP top candidate size (139.5 30.8 nm).

We next measured the percent encapsulation efficiency (EE) of DiD dye inside NPs coated with the top IL candidates (Fig.1B & Table S4) to be from 42.2 11.4 (CA3None 1:2) to 67.7 5.1% (CA2PE 1:1), consistent with the EE of our published CA2HA 1:2 IL-NP control at 60.43 2.03% ^50^. It is possible that the EE variation arises from the impact of a water solvation layer between PLGA and the coating, which is dependent on the anion structure and its degree of hydrophobicity, as it assembles with the cation, which creates the primary interface on the PLGA surface ^57-62^.

### Structural composition directs engineered IL-PLGA NPs to drive different cellular membrane affinity in whole BALB/c mouse and human blood without inducing significant phagocytosis.

**Figure 2:**
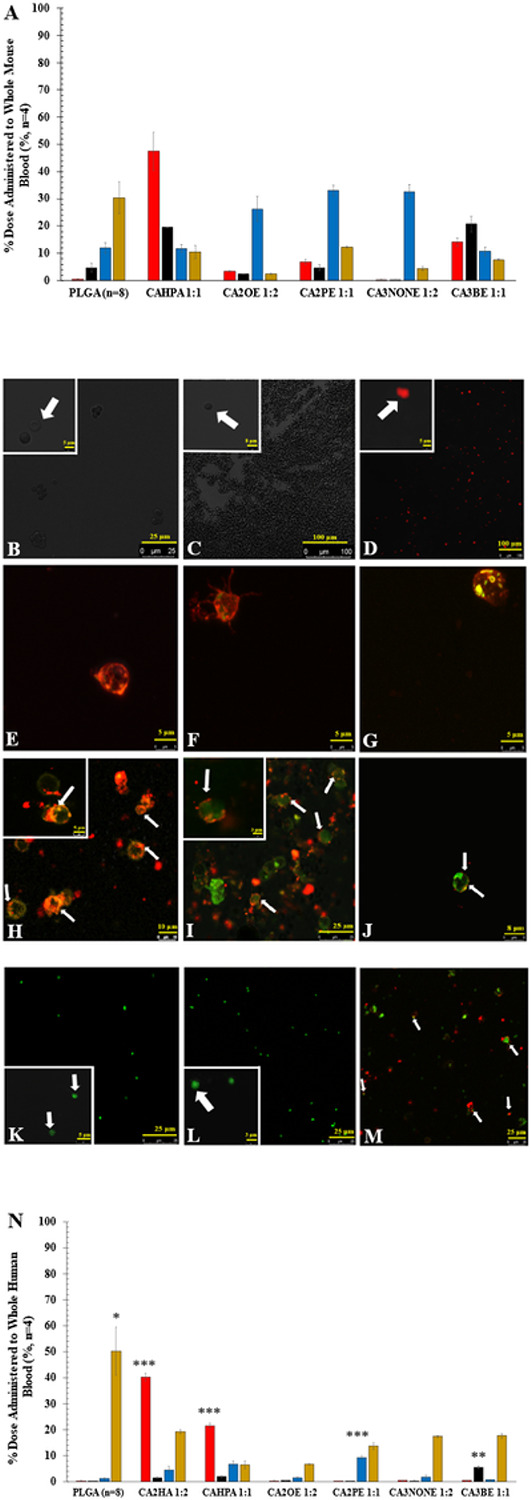
IL-PLGA DiD NP hitchhiking candidates show prominent qualitative and quantitative cellular hitchhiking in whole mouse blood via live-cell confocal microscopy (37 °C; yellow: *PE-CDllb*+, green: FITC-Ly6G+, red: DiD NPs) and fluorescent plate reader, which translates modestly into human whole blood via generally reduced serum residence (gold) compared to PLGA, and significantly increased fluorescence in RBC (red), platelet (black), and WBC populations (blue). **A)** Demonstrates cellular affinity of each top IL-PLGA DiD NP candidate in whole BALB/c mouse blood by fluorescent plate reader. Shown: quantified average % NP distribution per cell type for each top candidate in whole mouse BALB/c blood (n=4, SEM). Color key: Gold (Serum) , Blue (WBC), Black (Platelet), Red (RBC). Pictured by live cell confocal microscopy are the following isolated live cell populations (zoomed-in insets in white boxes) from whole blood treated with: **B)** 1x PBS-treated RBCs, **C)** PLGA-treated RBCs, **D)** CAHPA 1:1-treated RBCs; **E)** PLGA-treated Lymphocyte WBCs, **F)** PLGA-treated Dendritic Monocyte WBCs, **G)** PLGA-treated Granulocyte WBCs; **H)** CA2OE 1:2-treated Lymphocyte WBCs, **I)** CA2PE 1:1-treated Monocyte WBCs; **J)**CA3None 1:2-treated Granulocyte WBCs; **K)** 1x PBS-treated platelets, **L)**PLGA-treated platelets, **M)** CA3BE 1:1-treated platelets. **N)**Demonstrates translational cellular affinity of each top IL-PLGA DiD NP candidate (n=4) vs. bare PLGA (n=8) in human whole blood. Significance against bare PLGA was performed as a two-tailed t-test of unequal variance, with serum uptake screened across all candidates and bare PLGA via ANOVA (*** = p<0.001, ** = p<0.01, & * = p<0.05).

After FACS screening, top candidates were selected (Fig.1C) and the experiment was repeated using a fluorescent plate reader for quantitative evaluation *in situ* (n=4, SEM) as a percentage of cell-bound DiD-encapsulated NPs relative to amount mixed with whole BALB/c mouse blood (Fig.2A). It was revealed that, using a 1 x PBS-treated control baseline (Fig.S4), bare PLGA NPs were primarily found in the serum (30.4 ± 5.9%) with the next highest population likely phagocytosed by WBCs (11.9 ± 2.0%). In contrast, choline heptanoate 1:1 (CAHPA 1:1) bound nearly half of available RBCs (47.5 ± 7.0 %), choline 2-octenoate 1:2 (CA2OE 1:2) preferentially bound WBCs (lymphocytes, from the FACS screen) (26.3 ± 4.6 %), choline 2-pentenoate 1:1 (CA2PE 1:1) preferentially bound monocyte WBCs (33.1 ± 2.0 %), and choline 3-nonenoate 1:2 (CA3None 1:2) preferentially bound granulocyte WBCs (32.7 ± 2.6 %). Lastly, choline 3-butenoate 1:1 (CA3BE 1:1) favored platelets (20.8 ± 2.8 %) with some residual secondary affinity to RBCs (14.1 1.5%). Subsequently, all top candidates quantitatively evaluated in Fig.2A were immediately imaged by live-cell confocal microscopy to verify if the favored cellular populations bound were undergoing phagocytosis vs. hitchhiking (Fig.2B-M). Note that for each event of WBC cellular hitchhiking among top candidates (Fig.2.H-J), DiD colocalization (red) was clearly observed attaching/embedding into the membrane (green/yellow) as opposed to activation & direct cellular internalization observed by bare PLGA NPs (Fig.2.E-G). Additionally, the observed morphology of CA3BE 1:1-bound platelets suggested they were in a dormant state as opposed to activated, indicating stealth-like properties during hitchhiking.

Translational affinity of cellular hitchhiking was then evaluated by re-measuring quantitative distribution of the NP dose to whole blood in adult human female whole blood (n=4, SEM). As human whole blood demonstrates higher immunogenicity than mouse whole blood, bare PLGA NPs showed negligible affinity/uptake to other cell types and instead significantly enhanced residence in the serum (50.30 9.3%, ANOVA, p = 0.000128), likely from serum protein adsorption. Interestingly, positive control CA2HA 1:2 only slightly decreased in RBC hitchhiking capabilities in human whole blood (40.2 1.5 %), although with increased serum uptake (19.1 0.8%), indicating some weakened resistance in human whole blood. In contrast, top RBC candidate CAHPA 1:1 demonstrated half its RBC hitchhiking potential (21.4 1.04%) with drastically reduced serum uptake (6.41.5%) in human blood. Compared to platelet candidate CA3BE 1:1 (p = 0.0025, 5.3 0.5%) & monocyte candidate CA2PE 1:1 (p = 2.3 x , 9.3 0.6 %), a non-significant increase was seen in binding for lymphocyte candidate CA2OE 1:2 (1.4 0.2%) and granulocyte candidate CA3None 1:2 (1.6 0.8%). However, the lowered rates of serum uptake indicates that in human blood these NP candidates are more likely to free-circulate, instead of becoming directly opsonized. Importantly, to illustrate both translational affinity in human, and also to show that choice of anticoagulation (k_2_EDTA) was not artificially hindering phagocytosis, experimentation was replicated with heparin anticoagulant, and both BALB/c and human WBCs were then imaged by live-cell confocal microscopy, successfully indicating varying degrees of membrane penetration as opposed to phagocytic internalization (Fig. S2 & S3).

### Hitchhiking by engineered IL-PLGA NPs operates with stealth and biocompatibility in both whole BALB/c mouse and human blood.

To establish that cellular affinity was not inducing cytotoxicity in both BALB/c and human whole blood during the hitchhiking process, cellular biocompatibility was measured for RBCs (hemolysis), WBCS (PI/Annexin V), RBCs, and platelets (%CD62P+) by either plate reader or flow cytometry (FACS). Including CA2HA 1:2-coated reference NPs, all hitchhiking candidates remained under 10% hemolysis, exhibiting general RBC biocompatibility across human and mouse for IV injection. However, each IL-coated candidate interestingly exhibits different hemolytic properties that may be aligned to cell-type and cell-affinity-related variance in hitchhiking properties across platforms. For example, while CAHPA 1:1 (p = 0.88) shows negligible differences in hemolytic properties across mice and human RBCs, CA2PE 1:1 (p = 0.0014) and CA3None 1:2 (p = 0.00087) candidates showed significantly less hemolysis in human vs. mouse, as opposed to bare PLGA (p = 0.034), CA3BE 1:1(p = 0.02), and CA2OE 1:2-coated NPs (p = 3.8 x 10^−6^), which show significantly more hemolysis in human vs. mouse blood.

While all BALB/c and human WBCs show a total apoptosis in the range of under 10%, indicating high biocompatibility, the pattern of apoptosis differs between mouse and human lymphocytes, monocytes, and granulocytes. BALB/c lymphocytes show more late apoptosis than early, albeit no difference across treatments in both early (p = 0.054, ANOVA) or late apoptosis (p = 0.89, ANOVA). BALB/c monocytes instead show the opposite trend, additionally with a significant difference in only early apoptosis (p = 0.0066, ANOVA), occurring specifically between PLGA & CA3None 1:2 (p = 0.0077, two-tail t-test), but not across all treatments during late apoptosis (p = 0.17, ANOVA). Interestingly, bare PLGA NPs demonstrated significantly higher granulocyte early apoptosis than all other treatments (p = 8.4E-05, ANOVA), likely from both short-range electrostatic adsorption^63^ as well as phagocytic activity^64^ in response to the PLGA. During late granulocyte apoptosis, IL-coated NPs demonstrated generally lower levels compared to controls (p = 9.9 x 10^−6^, ANOVA) with granulocyte candidate CA3None 1:2-coated NPs notably demonstrating significant protective effects compared to bare PLGA (p = 0.0062, two-tail t-test). In contrast, human WBCs show negligible levels of late apoptosis across all treatments for lymphocytes (early: CA2PE 1:1 with the least relative to PLGA, p = 0.012; late:CA2PE 1:1 with the most relative to PLGA, p = 0.016) , monocytes (ANOVA early: p = 0.06; ANOVA late: p = 0.14) and granulocytes (CA2PE 1:1 with the least relative to PLGA: early (p = 0.027) & late (p = 0.03)), with a common trend of apoptosis occurring during the early phase.

Lastly, platelet activation was measured on the percentage of both BALB/c pooled gender and human adult female CD41+ platelets that co-stained for CD62P+ by FACS. Compared to BALB/c platelets, human platelets were found to naturally tend towards aggregation and activation when treated with the Adenosine Diphosphate (ADP) platelet activation positive control alone (two-tail t-test of means, p = 0.00026). Given this, the significance of platelet activation between mouse and human after bare PLGA NP treatment (p = 0.004), is surprising when directly contrasted with the lack of significant difference from CA3BE 1:1-coated PLGA NP treatment (p = 0.17). This finding not only confirms the presence of the IL coating on the PLGA NPs, but also indicates that membrane attachment facilitated by CA3BE 1:1 is performed without G-protein-coupled receptor surface activation^65-67^. Instead, the 3-Butenoic acid anion on the outermost layer of the PLGA coating is likely instead either noncovalently tethering/hydrogen-bonding to platelet membrane glycoproteins, to or within amino/choline phospholipid rafts, or engaging with flippase-mediated pump transport into the membrane ^68-71^. Additionally, because phospholipid-transport pathways are also present in RBCs, the mechanism of CA3BE 1:1 transport into the membrane may also regulate its secondary affinity to RBCs ^72,73^.

### Hitchhiking of RBCs, WBCs, and platelets by engineered IL-PLGA NPs in BALB/c mice significantly extends circulation half-life in vivo and drives dramatically altered biodistribution 24 hours post tail-vein injection.

The 24-hour pharmacokinetic and biodistribution profiles of the top IL-PLGA DiD NP candidates were evaluated in adult, healthy female BALB/c mice after intravenous tail-vein injection (approximate dosage in Table S3). No adverse physiological symptoms were observed for RBC, lymphocyte, granulocyte, or platelet candidates. However, monocyte candidate CA2PE 1:1 demonstrated some bowel disruption, likely from micro/lymph vessel NP shear during the homeostatic influx of cell-hitchhiked nanoparticles into lymph nodes located in the GI tract (such as intestine and jejunum)^74,75^, as well as the possible added effect of enterohepatic recycling^76^, aggregating the majority of CA2PE 1:1 PLGA DiD NPs in the intestine and pancreas with minimal clearance (Fig.S5). However, as shown in Figure S5, circulating monocytes that are bound by CA2PE 1:1 first traffic to the intestines from the tail vein at 2 hours (2 hours: intestine: 35.1%, pancreas: 1.5%) **before** migrating to the pancreas, with additional brain accumulation at 24 hours (24 hours: intestine: 14.9%, pancreas: 34.0%, brain: 6.3%). However, minimal accumulation in the remaining blood circulating organs, including the liver and spleen, indicates that the monocytes are not being triggered for phagocytic clearance or replenishing of inflammatory hepatic monocytes^77,78^. It is possible that initial monocyte trafficking to the intestines represents homeostatic entry of the lymphatic vasculature to join resident macrophages^79^, with either eventual differentiation into resident macrophages in the pancreas, complemented by the induction of inflammatory reactions such as inflammatory bowel syndrome (IBS) or mesenteric lymphadenitis^80,81^, or even possibly acute pancreatitis^82,83^. As such, these possible phenomena is likely responsible for the death of one mouse at the 2-hour timepoint, and another at the 6-hour timepoint. Issues such as this can likely be resolved via a further dosing study, and this concern may not pose such an issue within a diseased mouse model, where the monocytes are being actively transported to disease sites.

Encouragingly, the remaining white blood cell (WBC) hitchhiking candidates demonstrate notable biocompatibility over the 24-hour circulation period (n=3, average of injected dose (%) ± standard error of the mean (SEM)). In contrast with the negligible pharmacokinetic (PK) behavior of bare PLGA NPs alone at 1 hour (0.5 ± 0.07%) and 24 hours (0.13 ± 0.02%), lymphocyte candidate CA2OE 1:2 demonstrated an initial drop in circulating NPs to 42.2 ± 1.2% (p = 0.0007) at 1 hour with 10.8 ± 2.1% (p = 0.037) retained in the bloodstream at 24 hours. Granulocyte candidate CA3None 1:2 fared similar to CA2OE 1:2 at both 1 hour (46.4 ± 4.1%, p = 0.39) and 6 hours (29.0 ± 5.7%, p = 0.67) but had a non-significant higher retention in the bloodstream at 24 hours than CA2OE 1:2 (18.6 ± 1.0%, p = 0.13). Complementing their similar circulation PK profile, the intestine is the primary biodistribution site for both CA2OE 1:2 (39.8 ± 4.3%) and CA3None 1:2 (66.5 ± 9.5%) at 24 hours –however, granulocyte candidate CA3None 1:2 is much more highly selective to the intestines (secondary accumulation in liver: 10.6 ± 1.4%) than CA2OE 1:2, which has a secondary accumulation in the spleen (12.0 ± 4.5 %), tertiary accumulation in the heart (6.8 ± 0.6%), and lastly the liver (6.6 ± 1.5%).

Post tail-vein injection, both NP-hitchhiked lymphocyte and granulocyte populations would likely initially traffic from circulation to the first available lymph nodes in the intestine (for example: via T-cell recruitment and B-cell homing vs. chemokine-driven stimulated CCR7-expressing membrane-hitchhiked neutrophils^84,85^. Interestingly, the route of lymphatic entry to the intestine for lymphocytes (high endothelial venules to Peyer’s patches) differs from that of mesenteric entry of monocytes (afferent lymphatic vessels), and GI-tract entry of granulocytes (L-selectin-mediated entry to lymph interstitium): this may be a significant determinant in the perceived similarity in PK, but difference in BD and physiological response ^86-91^.

Additionally, after encountering the first point of shear (intestinal/gut lymph nodes), CA2OE 1:2-hitchhiked lymphocytes can re-enter the bloodstream circulation via exit through the efferent lymph and shearing through endothelial-modulated postcapillary venules, at which point lymphocytes can migrate downstream and access the cardiovascular and circulatory system through the thoracic duct, as well as may more easily activate, and enter the spleen, both of which were observed notable accumulation sites^92-94^. However, the vast majority of CA3None 1:2-coated NPs were observed to primarily remain in the intestines at 24 hours. The noticeable lack of travel to any other tissue may be due to weaker forces of membrane adhesion, potentially from the bulkiness of the anionic structure, allowing the IL-PLGA NPs to immediately shear onto endothelial cell linings at the first point of gut lymph entry from the tail-vein (with likely the remaining ~11% to be cleared from the liver) ^27^. However, the placement of the double bond at the anion’s tertiary carbon offers structural rigidity, which may offer protein-phobic protection (by sterics and reduced hydrogen-bonding) of sheared CA3None 1:2-PLGA NPs from further tissue clearance ^95^.

**Figure 4:**
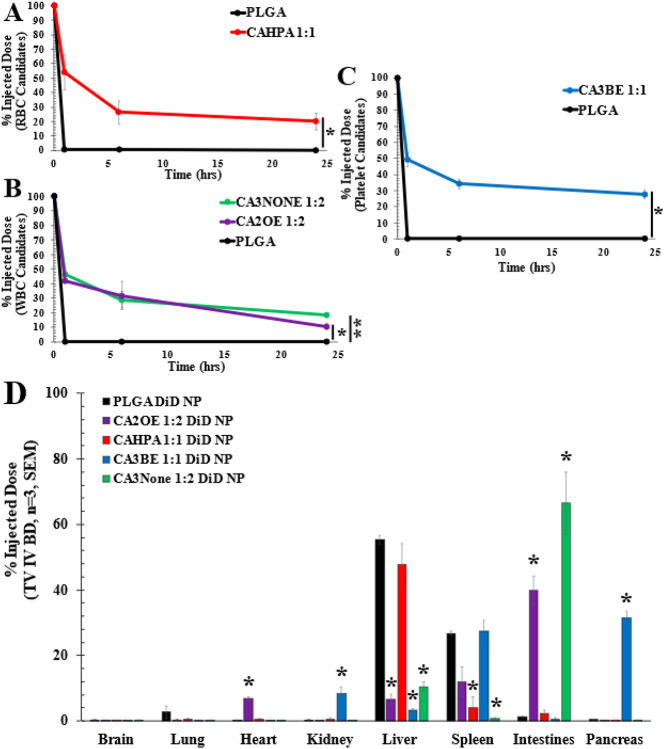
IL-PLGA NP candidates with selective cellular affinity in whole blood significantly extend BALB/c mouse pharmacokinetics (PK) and redirect organ biodistribution (BD) 24 hours after IV-tail-vein injection. **A-C)** PK curves & **D)** BD represented as average (n=3) of injected dose ± SEM. Bare PLGA NPs (black) functionalized at the surface with only carboxylic acid (black) are almost completely cleared from circulation 1 hour after injection and mainly accumulate in the liver & spleen. In contrast, all candidates have significantly higher retention at 24 hours, with WBC candidates bearing similar PK profiles to a PEGylated control^49^. Two-samples evaluated at a time via paired two-tail t-test):*=p<0.05, **=p<0.01. BD: Lymphocyte candidate CA2OE 1:2 (purple) and granulocyte candidate CA3None 1:2 (green) primarily accumulate in the intestine lymphatic vasculature, while platelet candidate CA3BE 1:1 (blue) redirects BD to the pancreas and spleen. RBC candidate CAHPA 1:1 (red) shears selectively in the liver, but with negligible accumulation in the spleen compared to bare PLGA. For BD, * indicates significantly less or greater organ accumulation at p<0.05 relative to bare PLGA NPs (black) per organ type.

In comparison, red blood cell candidate CAHPA 1:1 and platelet candidate CA3BE 1:1 both demonstrated similarly robust circulation profiles. CAHPA 1:1 was observed to reduce only about 50 % at 1 hour (53.9 ± 12.2%) and then taper to a stabilized retention of 20.2 ± 5.8% at 24 hours. Its initial drop and plateau between 6-24 hrs notably differs from the established PK profile of CA2HA 1:2, which had higher retention at every timepoint but a steeper decreasing curve. The structural difference between trans-2-hexenoic acid (2HA) and heptanoic acid (HPA) anions may play several roles in their IL-coating PK variation, as well as their observed BD (Fig.4D). Firstly, the alkyl chain of HPA is longer than 2HA by one carbon and is not restricted (6 free carbon centers) at the center of the chain by a trans double bond (3 free carbon centers). Because of this, HPA has more flexibility in the length of its free interacting alkyl chain tail, which potentially contributes different strengths in initial docking interactions with choline-rich phospholipids and the bilayer within the RBC membrane than 2HA, increasing the number of NPs adsorbed per RBC^96,97,31^. Additionally, because of the anionic structural difference, the hitchhiking pathway post-docking may also be different; for example, 2HA may interact with the short/medium chain monocarboxylate transport pathway^98-100^, while HPA may interact with the Band-3/Anion Exchanger (AE1) pathway ^101-104^.

When examining the biodistribution of CAHPA 1:1, 47.7 ± 6.4% accumulated in the liver, but only 4.2 ± 3.3% was directed to the spleen, far less than bare PLGA NPs (26.8 ± 0.65%). This suggests not direct protein-avoidance but stronger RBC membrane hitchhiking to avoid opsonization. This is because the first site of accumulation is not a gradual distribution of shear within chambers of the first pulmonary capillary bed (CA2HA 1:2), but likely a sudden NP “drop-off” of oxygenated RBCs from the lung circulation into the hepatic artery at 1 hour after IL-NP:RBC weakening from enduring multiple levels of matrix stiffness, shear stress, and arterial pressure^105-109^. Additionally, the location of liver accumulation is likely in the parenchyma and portal triad ducts, as some enterohepatic recycling from the liver to the bloodstream and intestines was observed (2.4 ± 1.1%), without further CAHPA 1:1 PLGA NP loss from circulation 6-24 hours^13^.

Despite a similar circulation PK profile to CAHPA 1:1 at 1 hour (p = 0.67), 6 hours (p = 0.26), and 24 hours (p = 0.11), CA3BE 1:1 is better retained in circulation at all timepoints over 24 hours in line with platelet circulation (24 hrs: 27.8 ± 3.0%)^110^. Respectively, the primary tissue accumulation sites of the platelet vs. RBC candidate was found to drastically differ. CA3BE 1:1-coated PLGA NPs were the only treatment that accumulated in the pancreas at 24 hours (31.4 ± 2.0%). Equivalent accumulation was also found in the spleen (27.5 ± 3.3%), with tertiary accumulation in the kidneys (8.5 ± 2.0%). Interestingly, however, and opposite to CAHPA 1:1, very little accumulated in the liver (3.2 ± 0.5%). With regards to the second site of accumulation, the spleen is a central organ responsible for platelet storage and driving interactions such as dynamic exchange with circulating platelets in the bloodstream^111-114^. This accumulation profile may also be partially contributed to by the secondary affinity to RBCs where the NP-surface binding density is much lower/weaker than CAHPA 1:1^115,116^. Rather than direction to clearance, pancreas accumulation may then reasonably follow from the splenic artery which shuttles blood to vascularize the pancreas from at least 4 entry sites^117^.

While the precise mechanism of biodistribution (ie. exact NP accumulation sites within tissue-specific cells) and anion-mediated cellular binding on a molecular level are still the subject of active investigation, this technology can be directly harnessed to use WBCs, RBCs, and platelets and access organs with high bioavailability and bioactivity for applications such as intestinal diseases (IBS, polyps, Crohn’s), pancreatic cancer, diabetes, sickle cell disease, renal disease, and liver diseases.

## Conclusions

Herein we describe, for the first time, biological ionic liquids that can be used as biocompatible polymeric nanoparticle coatings to interface with and selectively hitchhike onto different blood components *in situ*. While hitchhiking-mediated PK and BD are subject to change under disease conditions, which are not yet known^118^, this mode of cellular hitchhiking demonstrates the principle that the tissue locations of nanoparticle accumulation can be drastically redirected by structurally engineering the ionic liquid composition comprising the PLGA NP surface coating.While fundamental understanding of ionic liquid-membrane interactions is yet to be elucidated, the ability to target specific subpopulations of the blood across both mouse and human platforms allows for its use in a broad range of technologies, including immunotherapies and gene therapies. As such, this work opens the field towards further chemical, biological, and physical exploration of *in situ* cellular hitchhiking.

## Figures and Tables

**Figure 3:**
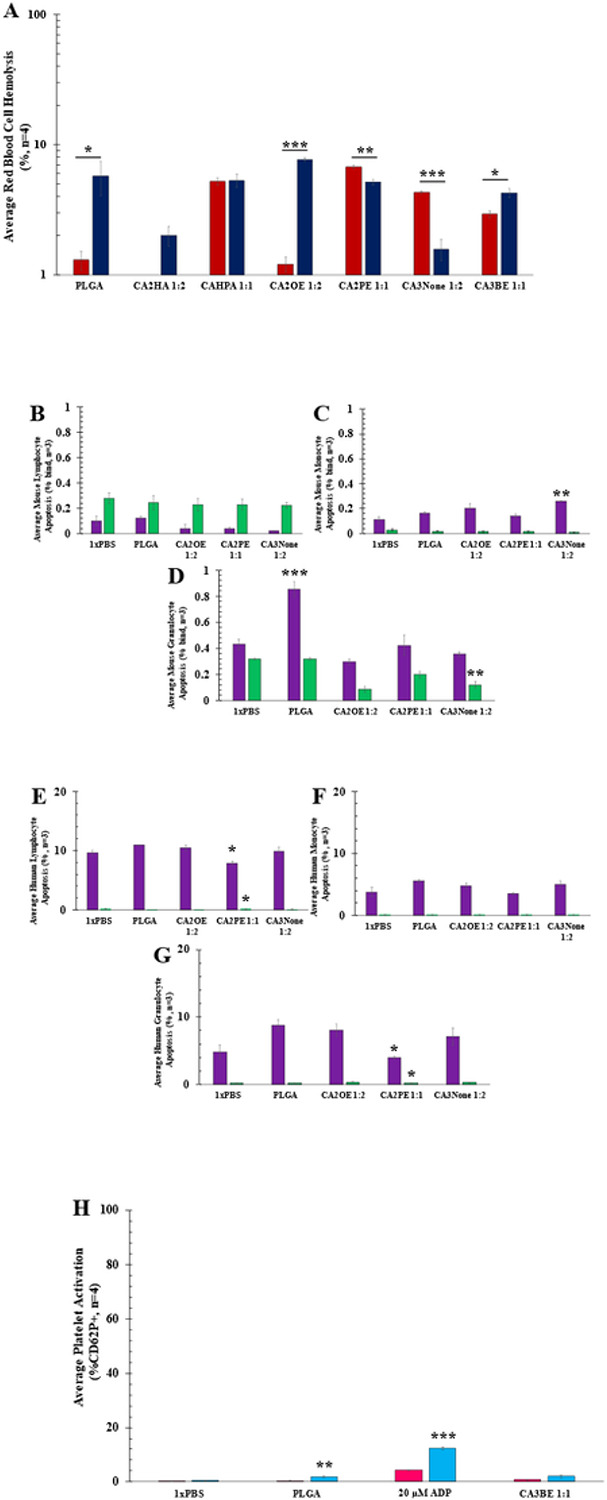
IL-PLGA NP candidates show high levels of biocompatibility with their respective cellular-hitchhiking affinity-types via RBC hemolysis, WBC apoptosis, and platelet activation assays. Data represented as average with standard error of mean (SEM). Significance determined by paired two-tailed t-test for two samples at a time between mouse and human samples: *=p<0.05, **=p<0.01, ***=p<0.001. **A)** Red Blood Cell Hemolysis Assay by fluorescent plate reader of all top candidates and bare PLGA NPs in BALB/c mouse RBCs (red) and Human RBCs (navy) (n=4, SEM). **B-G)** PI/Annexin V WBC early (purple) vs. late (green) apoptosis assayed by FACS of top lymphocyte (CA2OE 1:2), monocytes (CA2PE 1:1), and granulocyte (CA3None 1:2) hitchhiking candidates from: **B-D)**whole BALB/c mouse blood or **E-G)** whole human blood treatment (n=3, ± SEM). **H)** Platelet activation response by FACS (% activation, CD62P+ from CD41-labeled platelets) to platelet candidate CA3BE 1:1 in whole BALB/c mouse (pink) or human (blue) whole blood (n=4, SEM).
